# Bioactive adrenomedullin and interleukin-6 in COVID-19: potential biomarkers of acute kidney injury and critical illness

**DOI:** 10.1186/s12882-024-03486-1

**Published:** 2024-02-09

**Authors:** Simon B. Leininger, Stephan T. Staudner, Manuel J. Vogel, Julian Mustroph, Ute Hubauer, Stefan Wallner, Petra Lehn, Ralph Burkhardt, Christine Meindl, Frank Hanses, Markus Zimmermann, Lars S. Maier, Julian Hupf, Carsten G. Jungbauer

**Affiliations:** 1 Department of Internal Medicine II, University Hospital Regensburg, Franz-Josef-Strauss-Allee 11, 93053 Regensburg, Germany; 2 Department of Clinical Chemistry and Laboratory Medicine, University Hospital Regensburg, Franz-Josef-Strauss-Allee 11, 93053 Regensburg, Germany; 3 Emergency Department, University Hospital Regensburg, Franz-Josef-Strauss-Allee 11, 93053 Regensburg, Germany; 4 Department of Infection Prevention and Infectious Diseases, University Hospital Regensburg, Franz-Josef-Strauß-Allee 11, 93053 Regensburg, Germany

**Keywords:** COVID-19, SARS-CoV-2, Adrenomedullin, Interleukin-6, Acute Kidney Injury

## Abstract

**Background:**

The aim of this study was to investigate whether bioactive adrenomedullin (bio-ADM) and interleukin-6 (IL-6) are related to acute kidney injury (AKI) and severe illness in COVID-19 patients.

**Methods:**

153 patients with COVID-19 admitted to the emergency department (ED) were included. Blood samples were collected from each patient at admission. Bio-ADM and IL-6, as well as DPP3 and routinely measured markers were evaluated regarding the endpoints AKI (22/128 hospitalized patients) and a composite endpoint of admission to intensive care unit and/or in-hospital death (*n* = 26/153 patients).

**Results:**

Bio-ADM and IL-6 were significantly elevated in COVID-19 patients with AKI compared to COVID-19 patients without AKI (each *p* < 0.001). According to ROC analyses IL-6 and bio-ADM had the largest AUC (0.84 and 0.81) regarding the detection of AKI.

Furthermore, bio-ADM and IL-6 were significantly elevated in COVID-19 patients reaching the composite endpoint (each *p* < 0.001). Regarding the composite endpoint ROC analysis showed an AUC of 0.89 for IL-6 and 0.83 for bio-ADM in COVID-19 patients.

In the multivariable logistic model bio-ADM and IL-6 presented as independent significant predictors regarding both endpoints AKI and the composite endpoint in COVID-19 patients (as well as creatinine regarding the composite endpoint; each *p* < 0.05), opposite to leukocytes, C-reactive protein (CRP) and dipeptidyl peptidase 3 (DPP3; each *p* = n.s.).

**Conclusion:**

Elevated levels of bio-ADM and IL-6 are associated with AKI and critical illness in patients with COVID-19. Therefore, both biomarkers may be potential tools in risk stratification in COVID-19 patients at presentation in the ED.

**Supplementary Information:**

The online version contains supplementary material available at 10.1186/s12882-024-03486-1.

## Introduction

SARS-CoV-2, which was first detected in Wuhan, China, in late 2019, is the cause of COVID-19. Due to its contagious properties, it triggered a pandemic and continues to circulate as a viral cause of lung disease.

Even in countries with stable healthcare systems, such as Germany, COVID-19 continues to pose a major challenge. The consequences are perceptible in almost all areas of society, whether in economy, education, social interaction or personal relationships.

In most people, the virus induces a flu-like infection of the upper respiratory tract. However, the spectrum ranges from patients who experience no symptoms at all to very severe courses that require intensive medical treatment. In the worst case an infection has fatal consequences.

The pulmonary manifestations of SARS-CoV-2 are well known, however, it also affects other organs, such as the kidney. Thus, a frequent occurrence of acute kidney injury (AKI) has been observed in COVID-19 patients [1]. Moreover, the occurrence of AKI has been shown to be related to a more severe course [2, 3].

Early detection of renal impairment may thus represent an important role for early risk stratification in COVID-19 patients.

This will allow COVID-19 patients to receive the most appropriate therapy and to use limited medical resources as efficiently as possible.

In the current study, several biomarkers were evaluated for their predictive value regarding COVID-19 patients at admission in the Emergency Department.

Two of these are the biologically active form of adrenomedullin (bio-ADM) and interleukin 6 (IL-6):

Adrenomedullin (ADM) is a peptide hormone, that is involved in the regulation of vascular stability and permeability of blood vessels [4]. Further, ADM shows anti-inflammatory, anti-apoptotic and proliferative features [5].

Several studies demonstrated an association between elevated bio-ADM levels and unfavourable outcome in several diseases, i.e. patients with heart failure [6], cardiogenic shock [7] or sepsis [8–10]. In order to evaluate this value in COVID-19 patients, the focus of the current study was placed on bio-ADM.

IL-6 is a proinflammatory cytokine that has numerous effects, such as stimulation of immune cells, induction of acute-phase proteins and induction of fever [11]. An association between elevated IL-6 values and mortality was demonstrated in previous studies, for example in patients with sepsis [12], which is why the current study additionally focused on IL-6 in this comparison of biomarkers.

The aim of the study was to evaluate the prognostic value of bio-ADM and IL-6 regarding AKI or critical illness in COVID-19 patients and to compare them with DPP3 and routinely measured markers, making it the first study to our knowledge that investigates the prognostic value of bio-ADM compared to IL-6 measured at hospital admission in COVID-19 patients.

## Methods

Between March 2020 and June 2021, 243 patients presenting to the emergency department (ED) of a tertiary care hospital with symptoms of respiratory infection (i.e. fever, cough and/or dyspnea) were included. Patients under the age of 18 or not able to understand and sign the declaration of consent were excluded.

**Fig. 1:**
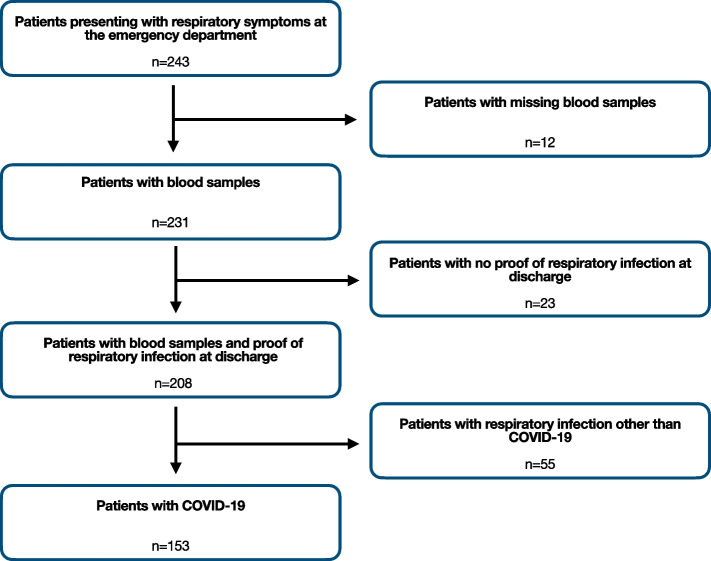
Stard flow diagram of the study population

Every patient was tested for SARS-CoV-2 with PCR analysis using throat rinse water or a nasopharyngeal swab. Patients with missing blood samples (*n* = 12), no proof of respiratory infection (*n* = 23) or respiratory infection other than COVID-19 (*n* = 55) were excluded (Fig. 1).

Baseline characteristics, vital signs and data of clinical course were documented for every included patient. For the statistic analysis of AKI only hospitalized patients were included, as no adequate data regarding their further clinical course could be obtained from the patients who remained ambulatory. AKI was assessed in accordance with KDIGO criteria from 2012 [13]. The second endpoint was a composite endpoint of admission to intensive care unit (ICU) and/or in-hospital death.

The study was approved by the Ethics Committee of the University of Regensburg and executed in accordance with Good Clinical Practice guidelines and within the standards established for human experimentation by the Declaration of Helsinki.

### Sample processing and laboratory analysis

Blood samples were collected in the ED immediately after admission, provided that consent was given and presuming that inclusion criteria were fulfilled. Routine markers such as IL-6, N-terminal brain natriuretic peptide (NT-proBNP), high-sensitivity cardiac troponin T (hsTnT), creatinine, cystatin c and C-reactive protein (CRP) were measured directly with devices from the company Roche Diagnostics Ltd. (Rotkreuz, Switzerland) at the central laboratory of the university hospital Regensburg.

Additionally, EDTA samples were centrifuged, aliquoted in 500 μl cups and frozen at -80 °C at the hospital laboratory. Frozen samples were thawed immediately before analysis. Bioactive adrenomedullin 1–52 (bio-ADM) was measured in EDTA plasma samples using the immunoluminometric sphingotest® assay (SphingoTec GmbH, Hennigsdorf, Germany) as described previously [14]. The 97.5th percentile in healthy adult subjects is 29 pg/mL (90% CI 27 – 38 pg/mL). The clinical cut-off for patients with sepsis and septic shock is 70 pg/mL [15, 8].

Circulating dipeptidyl peptidase 3 (DPP3) was measured in EDTA plasma samples by 4TEEN4 Pharmaceuticals (Hennigsdorf, Germany) using the immunoluminometric assay sphingotest® DPP3 (SphingoTec GmbH, Hennigsdorf, Germany) as described previously [16]. Based on the manufacturer’s instruction for use, the 97.5th percentile for sphingotest® DPP3 in healthy adult subjects is 22 ng/mL (90% CI 18 – 34 ng/mL). The clinical cut-off for critically ill patients is 40 ng/mL [17, 18].

The laboratory performing the biomarker measurement of bio-ADM and DPP3 was blinded to clinical and demographic data of the patients.

### Statistics

Baseline statistics were displayed with median (with interquartile range; IQR) or percentage.

For analysis of categorial variables Fisher’s exact test was used, for normally, continuous variables the student’s t-test and for non-normally distributed continuous values the Mann–Whitney-U test. Furthermore, receiver operating characteristic (ROC) analysis was performed. The area under the curve (AUC) of two or more ROC curves were compared using the methodology according to DeLong et al. [19]. Binary logistic regression was performed to detect independent predictors. Therefore IL-6, bio-ADM, DPP3, CRP, creatinine and white blood cell count were used. SPSS 25 (SPSS Inc., Chicago, Illinois) and MedCalc 20.019 (Mariakerke, Belgium) were used for statistical analysis.

## Results

### Study population

A total of 153 patients were included in the current study. Baseline characteristics regarding the overall collective are depicted in Table 1 and S1 and regarding hospitalized patients in Table 2 and S2.

**Table 1:** Baseline Characteristics: Vital signs and Biomarkers in the overall collective

	***Overall Collective***	***Composite endpoint***	***No composite endpoint***	***p composite endpoint vs. no composite endpoint***
***n***	*153*	*26*	*127*	
***Age (y)*** ^*a*^	*55.6* ± *15.7*	*64.8* ± *14.1*	*53.7* ± *15.4*	< *.001* ^*b*^
***Sex, % male*** ***(n)***	*56.2 (86)*	*65.4 (17)*	*54.3 (69)*	*.387* ^c^
***Vital signs at admission to ED***				
***Heart rate (b.p.m.)*** ^*a*^	*87* ± *15.7*	*94* ± *16.7*	*86* ± *15.1*	*.010* ^d^
***Systolic blood pressure (mmHg)*** ^*a*^	*130* ± *19.3*	*135* ± *28.0*	*128* ± *16.9*	*.096* ^d^
***Diastolic blood pressure (mmHg)*** ^*a*^	*82* ± *15.7*	*84* ± *26.0*	*81* ± *12.8*	*.374* ^d^
***Oxygen demand, %*** ***(n)***	*45.1 (69)*	*76.9 (20)*	*38.6 (49)*	< *.001* ^c^
***Respiratory rate (/min)*** ^*a*^	*22* ± *6.0*	*26* ± *6.2*	*21* ± *5.6*	< *.001* ^d^
***NEWS-2*** ^*e*^	*4 (1.5–6)*	*7 (5.75–9)*	*3 (1–5)*	< *.001* ^*b*^
***Biomarkers***				
***bio-ADM (pg/ml)*** ^*e*^	*34.5 (22.5–52.5)*	*58.8 (40.2–104.1)*	*30.4 (21.3–43.6)*	< *.001* ^*b*^
***IL-6 (pg/ml)*** ^*e*^	*32.9 (15.1–69.7)*	*107.5 (67.4–241.3)*	*26.5 (13.1–50.2)*	< *.001* ^*b*^
bio-ADM*IL-6 (pg/ml x pg/ml) ^*e*^	*1108.3 (432.8–3029.6)*	*7243.7 (2443.5–32,699.1)*	*853.2 (333.0–2062.6)*	< *.001* ^*b*^
***Serum creatinine (mg/dl)*** ^*e*^	*0.89 (0.75–1.22)*	*1.05 (0.85–1.42)*	*0.87 (0.72–1.17)*	*.014* ^*b*^
***dpp3 (ng/ml)*** ^*e*^	*19.2 (14.5–30.6)*	*34.8 (20.8–51.3)*	*17.8 (13.2–26.9)*	< *.001* ^*b*^
***hsTnT (ng/l)*** ^*e*^	*8.5 (5.1–14.3)*	*16.8 (9.8–54.5)*	*6.7 (4.3–12.1)*	< *.001* ^*b*^
***NT-proBNP (pg/ml)*** ^*e*^	*74.4 (50.0–311.0)*	*455.5 (86.4–2367.75)*	*67.0 (50.0–191.0)*	< *.001* ^*b*^
***White blood cell count (n/nl)*** ^*e*^	*5.53 (4.11–7.49)*	*6.98 (4.76–9.78)*	*5.12 (3.93–7.22)*	*.013* ^*b*^
***CRP (mg/dl)*** ^*e*^	*42.4 (18.6–90.4)*	*112.0 (74.0–163.0)*	*32.0 (15.5–70.0)*	< *.001* ^*b*^
***Procalcitonin (ng/ml)*** ^*e*^	*0.08 (0.06–0.15)*	*0.25 (0.09–0.52)*	*0.07 (0.06–0.12)*	< *.001* ^*b*^

^a^ Mean ± standard deviation

^b^ Mann–Whitney-U

^c^ Fisher’s exact test

^d^ Student’s t-test

^e^ Median (interquartile range)

**Table 2:** Baseline Characteristics: Vital signs and Biomarkers in hospitalized COVID-19 patients

	***Hospitalized COVID-19 patients***	***AKI***	***No AKI***	***p AKI vs. no AKI***
***n***	*128*	*22*	*106*	
***Age (y)*** ^*a*^	*58.1* ± *14.5*	*64.2* ± *14.7*	*56.8* ± *14.2*	*.031* ^*b*^
***Sex, % male (n)***	*57.0 (73)*	*68.2 (15)*	*54.7 (58)*	*.344* ^c^
*Vital signs at admission to ED*				
***Heart rate (b.p.m.)*** ^*a*^	*88* ± *15.9*	*94* ± *18.4*	*87* ± *15.1*	*.074* ^d^
***Systolic blood pressure (mmHg)*** ^*a*^	*130* ± *20.3*	*130* ± *23.3*	*130* ± *19.7*	*.960* ^d^
***Diastolic blood pressure (mmHg)*** ^*a*^	*82* ± *16.9*	*77* ± *17.3*	*83* ± *16.7*	*.177* ^d^
***Oxygen demand, % (n)***	*53.9 (69)*	*63.6 (14)*	*51.9 (55)*	*.355* ^c^
***Respiratory rate (/min)*** ^*a*^	*23* ± *6.0*	*26* ± *6.2*	*22* ± *5.8*	*.007* ^d^
***NEWS-2*** ^*e*^	*4 (2–6.75)*	*7 (4–9)*	*4 (2–6)*	< *.001* ^*b*^
***Biomarkers***				
***bio-ADM (pg/ml)*** ^*e*^	*37.8 (26.6–56.2)*	*60.0 (46.0–105.9)*	*35.0 (23.7–50.0)*	< *.001* ^*b*^
***IL-6 (pg/ml)*** ^*e*^	*39.5 (18.5–77.1)*	*111.0 (61.7–288.0)*	*33.9 (16.9–61.3)*	< *.001* ^*b*^
bio-ADM*IL-6 (pg/ml x pg/ml) ^*e*^	*1610.3 (560.0–3817.4)*	*9807.0 (2847.4–32,699.1)*	*1103.0 (502.1–2437.7)*	< *.001* ^*b*^
***Serum creatinine (mg/dl)*** ^*e*^	*0.93 (0.77–1.31)*	*1.18 (1.00–1.57)*	*0.88 (0.74–1.20)*	< *.001* ^*b*^
***dpp3 (ng/ml)*** ^*e*^	*21.5 (16.0–32.8)*	*31.6 (20.0–54.1)*	*19.4 (15.2–30.0)*	*.004* ^*b*^
***hsTnT (ng/l)*** ^*e*^	*9.2 (5.8–16.7)*	*26.3 (13.1–54.5)*	*8.6 (5.4–13.7)*	< *.001* ^*b*^
***NT-proBNP (pg/ml)*** ^*e*^	*104.5 (50.0–390.8)*	*699.0 (134.8–3144.0)*	*75.0 (50.0–291.3)*	< *.001* ^*b*^
***White blood cell count (n/nl)*** ^*e*^	*5.74 (4.15–7.96)*	*7.25 (4.99–11.71)*	*5.50 (4.02–7.43)*	*.010* ^*b*^
***CRP (mg/dl)*** ^*e*^	*52.7 (24.8–96.4)*	*117.5 (63.9–163.0)*	*44.9 (22.0–82.5)*	< *.001* ^*b*^
***Procalcitonin (ng/ml)*** ^*e*^	*0.09 (0.06–0.18)*	*0.30 (0.11–0.44)*	*0.08 (0.06–0.14)*	< *.001* ^*b*^

^a^ Mean ± standard deviation

^b^ Mann–Whitney-U

^c^ Fisher’s exact test

^d^ Student’s t-test

^e^ Median (interquartile range)

The average age of the overall cohort was 56 years and 56.2% patients were male. Most common symptoms at admission to ED were fatigue, cough, fever and dyspnea (Table S1).

Arterial hypertension was the most common pre-existing disease (40.5%), about 13% suffered from chronic kidney injury.

Patients who reached the composite endpoint were significantly older (*p* < 0.001); at admission to ED they showed a higher respiratory rate, NEWS-2 score and demand of oxygen (each *p* < 0.001).

Furthermore, coronary artery disease, chronic heart failure, obesity and arterial hypertension as pre-existing diseases were more common in patients who reached the combined endpoint (each *p* < 0.05).

Among the 128 hospitalized COVID-19 patients, the average age was 58 years.

Regarding the symptoms at presentation to the ED, there were no major differences in hospitalized patients who did develop AKI and those who did not (Table S2), but respiratory rate and NEWS-2 score were significantly higher in those with AKI (both *p* < 0.05).

Arterial hypertension, chronic kidney injury and coronary artery disease were also more common in hospitalized patients with AKI (each *p* < 0.05).

### Acute kidney injury in COVID-19 patients

For the evaluation of the endpoint AKI only the 128 hospitalized COVID-19 patients were enrolled. 22 patients suffered from AKI during the in-hospital stay. The median time between admission to ED and development of AKI was 5 days.

**Fig. 2:**
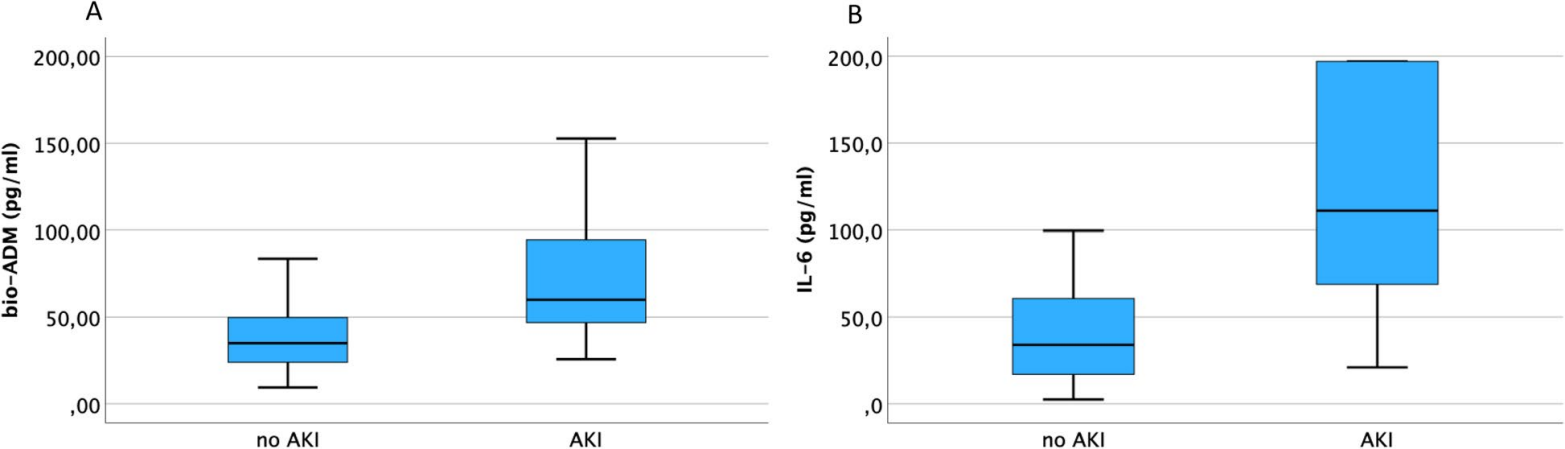
Boxplots: AKI in hospitalized patients

Bio-ADM and IL-6 were significantly elevated in patients with AKI compared to those without (each *p* < 0.001, Fig. 2), same as hsTnT, NT-proBNP, creatinine, CRP, DPP3, PCT and leukocytes (each *p* < 0.05).

**Fig. 3:**
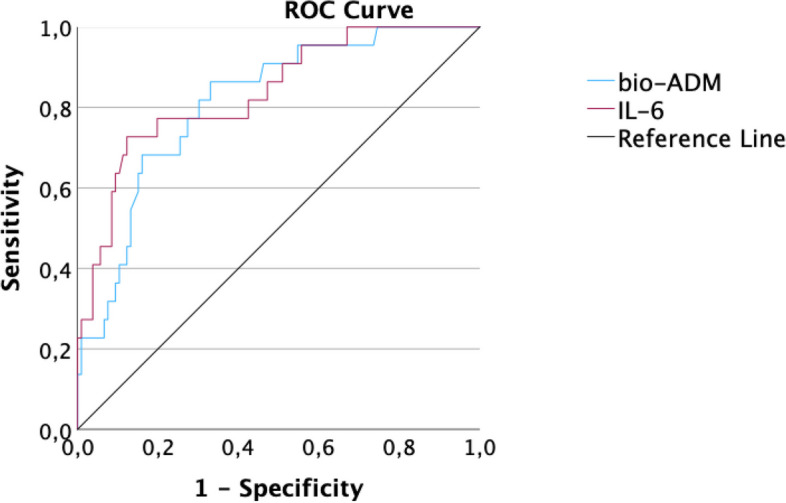
Receiver operating characteristic analysis of bio-ADM and IL-6 regarding the endpoint AKI in hospitalized patients

ROC analysis regarding the endpoint AKI showed an AUC of 0.84 for IL-6 and 0.81 for bio-ADM(Fig. 3), therefore having the highest discriminatory capability of all other biomarkers in this comparison like hsTnT, NT-proBNP and creatinine (0.80, 0.76 and 0.75). The ROC curve of IL-6 differed significantly from leukocytes and DPP3, but no other biomarkers in the panel.

The product of IL-6 and bio-ADM had the highest predictive value for development of AKI (AUC = 0.86), but did not have any significant added value in comparison to one of these two markers alone (*p* = n.s.).

In a univariate regression analysis bio-ADM and IL-6 were both significant predictors (OR 1.025, 95% CI 1.011–1.040, *p* < 0.001 and OR 1.014, 95% CI 1.005–1.023, *p* = 0.003) regarding acute kidney injury, same as CRP, hsTnT and DPP3 (each *p* < 0.05) and opposite to leukocytes, creatinine, procalcitonin and NT-proBNP (each *p* = n.s.).

In a multivariable logistic model bio-ADM (OR: 1.017, 95% CI 1.001–1.034, *p* = 0.036) and IL-6 (OR: 1.010, 95% CI 1.001–1.018, *p* = 0.027) were independent significant predictors regarding AKI, in contrast to creatinine, leukocytes, CRP and DPP3 (each *p* = n.s). (Table 3).

**Table 3:** Univariate and Multivariate logistic model for detection of AKI in hospitalized COVID-19 patients

	***Univariate***		***Multivariate***	
	***OR (95% Confidence Intervall)***	***P***	***OR (95% Confidence Intervall)***	***P***
***IL-6***	*1.014 (1.005–1.023)*	*0.003*	*1.010 (1.001–1.018)*	*0.027*
***bio-ADM***	*1.025 (1.011–1.040)*	< *0.001*	*1.017 (1.001–1.034)*	*0.036*
***Creatinine***	*1.440 (0.880–2.356)*	*0.147*	*0.861 (0.475–1.558)*	*0.620*
***DPP3***	*1.024 (1.003–1.045)*	*0.024*	*1.004 (0.976–1.032)*	*0.778*
***CRP***	*1.014 (1.006–1.022)*	< *0.001*	*1.000 (0.984–1.016)*	*0.975*
***Leukocytes***	*1.096 (1.000–1.202)*	*0.050*	*1.057 (0.953–1.172)*	*0.296*
***hsTnT***	*1.018 (1.003–1.033)*	*0.020*	*-*	
***NT-proBNP***	*1.000 (1.000–1.000)*	*0.733*	*-*	
***procalcitonin***	*1.405 (0.757–2.605)*	*0.281*	*-*	

### Composite endpoint: admission to ICU and/or in-hospital death in COVID-19 patients

Regarding the composite endpoint, 153 patients were included. 23 patients were admitted to the ICU and 13 patients died. The composite endpoint was reached by 26 patients. The median time between hospital admission and death was 14 days.

14 patients were admitted to the ICU on the same day as the presentation in the ED. Two patients were treated with ECMO (extracorporal membrane oxygenation).

**Fig. 4:**
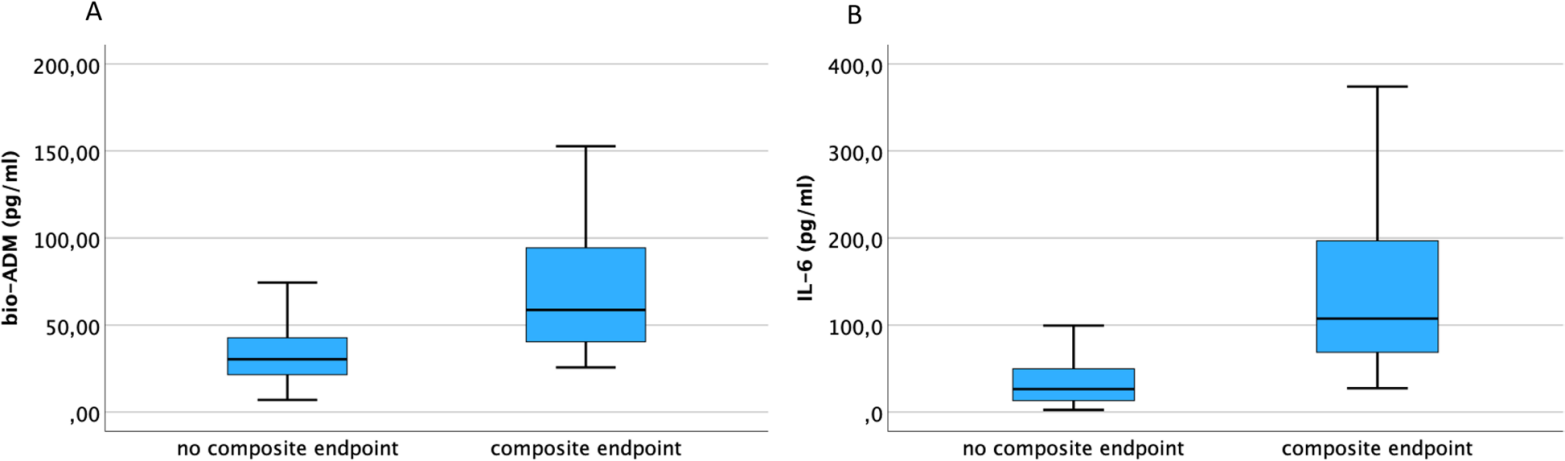
Boxplots: composite endpoint. Boxplots showing bio-ADM and IL-6 in COVID-19 patients who were admitted to the ICU and/or died during their in-hospital stay. A Bio-ADM is significantly elevated in COVID-19 patients who reached the composite endpoint compared to those who did not (p < 0.001). The Median of bio-ADM regarding COVID-19 patients who reached the composite endpoint and those who did not was 58.8 pg/nl and 30.4 pg/nl. B IL-6 is significantly elevated in COVID-19 patients who reached the composite endpoint compared to those who did not (*p* < 0.001). The Median of IL-6 regarding COVID-19 patients who reached the composite endpoint and those who did not was 107.5 pg/nl and 26.5 pg/nl

Bio-ADM and IL-6 were significantly elevated in patients reaching the composite endpoint (each *p* < 0.001, Fig. 4), same as hsTnT, NT-proBNP, creatinine, CRP, DPP3, procalcitonin and leukocytes (each *p* < 0.05).

**Fig. 5:**
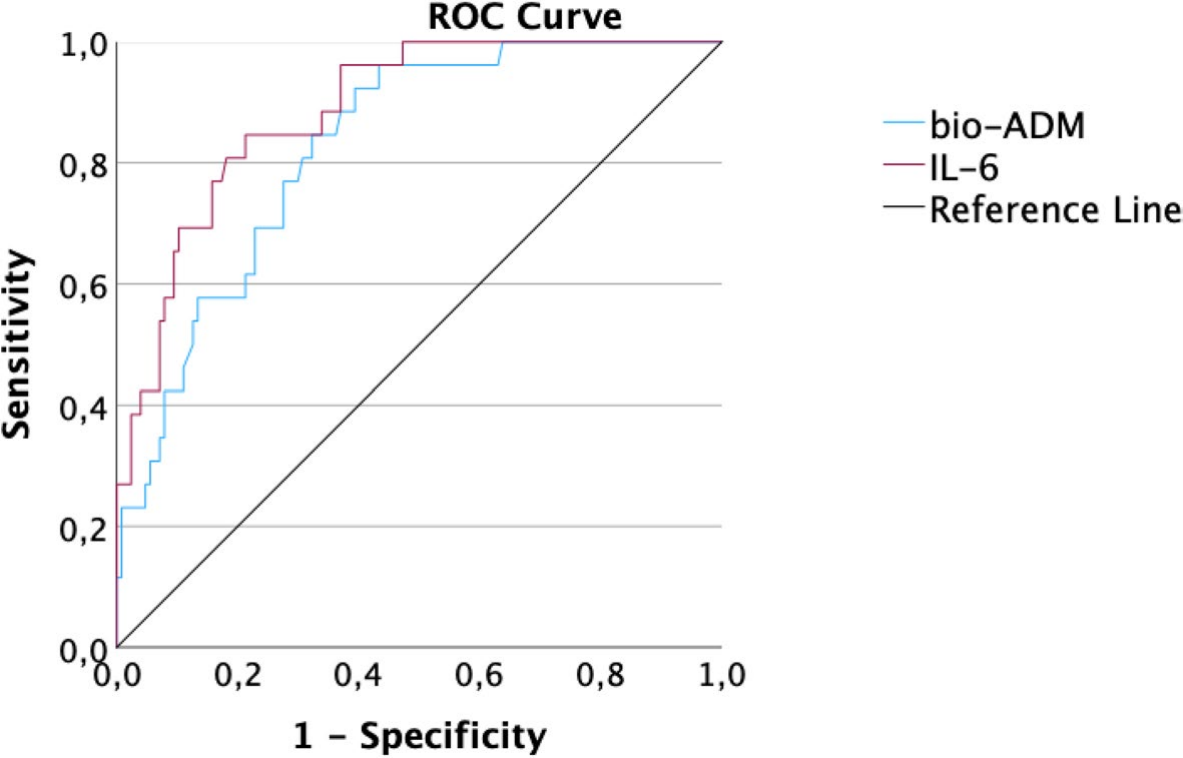
Receiver operating characteristic analysis of bio-ADM and IL-6 regarding the composite endpoint. Predictive values of bio-ADM (blue) and IL-6 (red) regarding the composite endpoint in COVID-19 patients. Area under the curve (AUC) bio-ADM: 0.83 (cut-off value of 38.7 pg/ml: sensitivity: 84.6%, specificity: 67.7%, *p* < 0.001). AUC IL-6: 0.89 (cut-off value 57.8 pg/ml: sensitivity: 84.6%, specificity: 78.7%, *p* < 0.001)

According to ROC analyses, IL-6 (AUC = 0.89) had the largest AUC regarding the prediction of the composite endpoint, followed by CRP (AUC = 0.83) and bio-ADM (AUC = 0.83, Fig. 5). HsTnT (AUC = 0.80), DPP3 (AUC = 0.80), procalcitonin (PCT; AUC = 0.79), NT-proBNP (AUC = 0.75), leukocytes (AUC = 0.66) and creatinine (AUC = 0.65) showed lower values. In comparison of ROC curves, bio-ADM differed significantly from creatinine, leukocytes and DPP3 (each *p* < 0.05). In addition to these three biomarkers IL-6 also differed significantly from procalcitonin (PCT) and NT-proBNP (each *p* < 0.05).

The product of IL-6 and bio-ADM (AUC = 0.90) showed the largest AUC, but did not have any significant added value in comparison to one of these markers alone (each *p* = n.s.).

In a univariate regression model, bio-ADM and IL-6 were both significant predictors (OR 1.029, 95% CI 1.015–1.044 and OR 1.023, 95% CI 1.013–1.033; both *p* < 0.001) regarding the endpoint critical illness, same as creatinine, CRP, DPP3, hsTnT and leukocytes (each *p* < 0.05) and opposite to procalcitonin and NT-proBNP (both *p* = n.s.).

In a multivariate logistic model IL-6 (OR: 1.021, 95% CI 1.010–1.033, *p* < 0.001), bio-ADM (OR: 1.021, 95% CI 1.003–1.040, *p* = 0.021) and creatinine (OR: 0.522, 95% CI 0.300–0.909, *p* = 0.022) were independent significant predictors in the COVID-19 cohort regarding the composite endpoint, in contrast to leukocytes, CRP and DPP3 (each *p* = n.s.) (Table 4).

**Table 4:** Univariate and Multivariate logistic model regarding the composite endpoint

	***Univariate***		***Multivariate***	
	***OR (95% Confidence Intervall)***	***P***	***OR (95% Confidence Intervall)***	***P***
***IL-6***	*1.023 (1.013–1.033)*	< *0.001*	*1.021 (1.010–1.033)*	< *0.001*
***bio-ADM***	*1.029 (1.015–1.044)*	< *0.001*	*1.021 (1.003–1.040)*	*0.021*
***Creatinine***	*1.376 (0.891–2.126)*	< *0.001*	*0.522 (0.300–0.909)*	*0.022*
***DPP3***	*1.033 (1.012–1.056)*	*0.002*	*1.016 (0.993–1.040)*	*0.172*
***CRP***	*1.022 (1.012–1.031)*	< *0.001*	*0.225 (0.994–1.024)*	*0.225*
***Leukocytes***	*1.095 (1.002–1.196)*	*0.045*	*0.965 (0.832–1.119)*	*0.639*
***hsTnT***	*1.023 (1.007–1.040)*	*0.004*	*-*	
***NT-proBNP***	*1.000 (1.000–1.000)*	*0.227*	*-*	
***procalcitonin***	*1.552 (0.785–3.066)*	*0.206*	*-*	

## Discussion

The aim of the current study was to assess the prognostic value of bio-ADM and IL-6 regarding AKI and critical illness (admission to ICU and/or in-hospital death) in patients with COVID-19. This is the first study to our knowledge that investigates the prognostic value of bio-ADM compared to IL-6 measured at hospital admission in COVID-19 patients.

In patients with COVID-19, bio-ADM and IL-6 were significantly elevated in patients suffering from acute kidney injury during their in-hospital stay as well as reaching the combined endpoint. Both markers were independent and significant predictors regarding both endpoints in a multivariate regression analysis. Other biomarkers, like procalcitonin, were no independent significant predictors for either endpoint in patients with COVID-19 in the multivariate logistic model (data not shown).

One of the biomarkers compared in the current study was DPP3, which was shown to be a significant independent predictor of SARS-CoV-2 infection in a previous study [20]. In the current study, DPP3 was elevated in both endpoints of the COVID-19 cohort, but in ROC analysis, bio-ADM and IL-6 each had a higher AUC than DPP3. DPP3 was also not found to be an independent significant predictor for any of the endpoints, which is why no further focus was put on DPP3 in this study (data not shown).

Thus, in conclusion, bio-ADM and IL-6 showed a high predictive value in patients with SARS-CoV-2 infection and may provide an important component in early risk stratification.

### Adrenomedullin in COVID-19

Adrenomedullin, as measured as bio-ADM in the current study, showed its prognostic value regarding severity of respiratory failure, need for extracorporal organ support and 28-day mortality [21] as well as development of AKI [22] in COVID-19 patients who were admitted to ICU, but these studies are not fully comparable to the current study, which included a broader spectrum of disease severity.

In a collective of 101 COVID-19 patients, Papasidero et al. evaluated bio-ADM at presentation to ED and found a significant predictive value regarding mortality, but not regarding to AKI, which might be due to the low number of patients with AKI in their collective [23].

In the present study, the high predictive value of bio-ADM in COVID-19 patients regarding a critical course can be confirmed. Furthermore, with the current findings and the higher number of endpoints, this predictive value can be extended to early detection of AKI, defined according to KDIGO, in COVID-19 patients.

In a proteome analysis of 177 different proteins, ADM was measured in 53 hospitalized COVID-19 patients. The assumption of a sicker collective than that of the current study is confirmed by a higher proportion of patients who were admitted to the ICU or died.

ADM was not significantly elevated in patients admitted to the ICU (*p* = n.s.), but was the strongest predictor of death in their comparison (*p* < 0.001, AUC = 0.87). [24].

Thus, in agreement with the present study, ADM is a potentially important predictor of critical outcome in COVID-19 patients. With the present study the predictive value may be extended to a less diseased population and the early detection of AKI.

An association between patients infected with SARS-CoV-2 and ADM, as measured by RNA expression of ADM, in a then smaller collective of 40 patients (21 COVID and 19 non-COVID patients), was published earlier by Hupf et al. [25]. In the COVID-19 cohort RNA expression of ADM showed significantly elevated values in patients who were admitted to the ICU. The present study confirms the prognostic value of ADM, in our former study measured by RNA expression and now as bio-ADM, regarding critical outcome in COVID-19 patients. The findings were examined in a larger collective and can be extended now to the endpoint AKI.

Previous studies demonstrated that elevated MR-proADM concentrations measured at admission to the ED correlate as well with mortality in COVID-19 patients [26–28].

Additionally, Indirli et al. demonstrated a significant correlation of elevated MR-proADM values and development of AKI in a COVID-19 cohort of 116 patients, however only 4 of them developed AKI [29].

Similar results were shown in the current study, highlighting the predicitve value of ADM and extending it to the measurement of bio-ADM. However, the exact stochiometric relationship between MR-proADM and bio-ADM is unclear [14].

### IL-6 in COVID-19

An association between elevated IL-6 values and bad outcome, for example regarding mortality in patients with sepsis [12], was demonstrated in previous studies, which provides the rationale for the focus of the current study on IL-6.

Regarding COVID-19, Ruan et al. found increased levels of IL-6 in patients with fatal outcome [30]. In that study, nearly half of the 150 patients deceased and in comparison to the present collective almost twice as many COVID-19 patients were admitted to the ICU. Therefore the patient collective is not fully comparable to the current study.

Regarding the early detection of patients that were later admitted to the ICU, Diao et al. reviewed a collective of 522 COVID-19 patients. IL-6 was found to be significantly elevated in the 43 patients admitted to the ICU [31].

Similar to these findings the present study supports the assumption that IL-6 may be a significant predictive biomarker for ICU admission or death in patients with COVID-19.

Furthermore, an association between elevated IL-6 values and development of AKI was demonstrated in COVID-19 patients who were admitted to the ICU [32]. The present study, which supports these findings, evaluated a larger, unselected collective and may thus extend the predictive value of IL-6 in detection of AKI to COVID-19 patients outside of ICU.

Additionally, in comparison to other biomarkers IL-6 was found to be a significant independent predictor regarding both endpoints in the present study, which emphasizes the high predictive value of IL-6 in COVID-19 patients.

### Relevance of bio-ADM and IL-6 in COVID-19

During the pandemic, many patients worldwide encountered limited healthcare system resources. Predicting the course of the disease as accurately and early as possible may play an important role in both health and economic terms. Early risk stratification may be an efficient means of being able to select adequate therapy at an early state of disease in the future.

The current study showed a high prognostic value in early detection of severe illness in COVID-19 patients for both IL-6 and bio-ADM, which is consistent with the results of previous studies.

Further, bio-ADM and IL-6 each were significantly elevated in patients with COVID-19, who reached either of the individual endpoints admission to the ICU or in-hospital death. According to the multivariate logistic model that was used in the present study, IL-6, bio-ADM and creatinine presented as independent significant predictors in COVID-19 patients regarding the endpoint admission to the ICU; regarding the endpoint in-hospital death only bio-ADM and CRP did present as independent significant predictors (data not shown).

Further, previous studies about IL-6 have shown a high predictive value in early detection of AKI in critically ill COVID-19 patients, whereas bio-ADM was correlated in only two studies to date regarding this endpoint.

According to the present study, both biomarkers have also shown a high predictive value in COVID-19 patients for the endpoint AKI, confirming their high potential in this patient collective.

Therefore, bio-ADM and IL-6 have high predictive value in COVID-19 patients at admission to the ED and thus can contribute to early risk stratification.

Bio-ADM did present as a significant predictor regarding the combined endpoint critical illness in COVID-19 patients. The present study highlights the potential predictive value of bio-ADM in general, which is subject of current investigation. However, further studies are needed to confirm the value of each marker and the combination of both markers in larger collectives.

### Limitations

The present study has several limitations. In this single center study, 153 COVID-19 patients were included, which is a rather small sample size.

Due to the small sample size, the findings must be regarded as hypothesis-forming. Larger studies are needed for validation.

In order to increase the number of patients reaching the endpoint critical illness and thus to increase the power of the study, a combination of admission to the ICU and in-hospital death was chosen. In larger studies, the composite endpoint could be split into two individual endpoints admission to the ICU and in-hospital death.

Also, due to the small population the number of variables in the multivariate analysis were limited.

Due to the small number of events in the evaluation of AKI, no attention could be paid to the subdivision in different KDIGO stages.

Furthermore, the present study covers patients enrolled up to June 2021. Therefore, later SARS-CoV-2 variants, such as delta and omicron, have not been included in our evaluation. Further studies with larger numbers of patients containing newer variants of SARS-CoV-2 are needed to confirm the results of this study.

### Supplementary Information

**Additional file 1: Supplementarly Table S1**. Further Baseline Characteristics regarding the overall collective

**Additional file 2: Supplementarly Table S2. **Further Baseline Characteristics regarding hospitalized patients.

## Data Availability

The datasets generated and analyzed during the current study are available from the corresponding author on reasonable request.
