# Supplementary material for: Bioactive adrenomedullin and interleukin-6 in COVID-19: potential biomarkers of acute kidney injury and critical illness
Source: BMC Nephrol. 2024 Feb 9;25:52. doi: 10.1186/s12882-024-03486-1 (PMC10858491; doi:10.1186/s12882-024-03486-1)
Supplement: Supplementary file 1 — Additional file 1: Supplementarly Table S1. Further Baseline Characteristics regarding the overall collective [file 12882_2024_3486_MOESM1_ESM.docx]

**Supplementarly Table S1: Further Baseline Characteristics regarding the overall collective**

|  | *Overall collective* | *Composite endpoint* | *No composite endpoint* | *p composite endpoint vs no composite endpoint* |
| --- | --- | --- | --- | --- |
| *n* | *153* | *27* | *126* |  |
| *Symptoms* |  |  |  |  |
| *Cough, % (n)* | *61.4 (94)* | *51.9 (14)* | *63.5 (80)* | *.387 ^a^* |
| *Dyspnea, % (n)* | *56.2 (86)* | *74.1 (20)* | *52.4 (66)* | *.029 ^a^* |
| *Fever, % (n)* | *56.9 (87)* | *40.7 (11)* | *60.3 (76)* | *.129 ^a^* |
| *Chills, % (n)* | *36.0 (55)* | *22.2 (6)* | *38.9 (49)* | *.179 ^a^* |
| *Sniffles, % (n)* | *11.8 (18)* | *7.4 (2)* | *12.7 (16)* | *.740 ^a^* |
| *Fatigue, % (n)* | *77.1 (118)* | *81.5 (22)* | *76.2 (96)* | *.444 ^a^* |
| *Anosmia, % (n)* | *17.0 (26)* | *11.1 (3)* | *18.3 (23)* | *.571 ^a^* |
| *Dysgeusia, % (n)* | *33.3 (51)* | *22.2 (6)* | *35.7 (45)* | *.261 ^a^* |
| *Pre-existing diseases* |  |  |  |  |
| *Coronary artery disease, % (n)* | *9.2 (14)* | *22.2 (6)* | *6.3 (8)* | *.016 ^a^* |
| *Chronic heart failure, % (n)* | *2.6 (4)* | *11.1 (3)* | *0.8 (1)* | *.016 ^a^* |
| *Arterial hypertension, % (n)* | *40.5 (62)* | *59.3 (16)* | *36.5 (46)* | *.027 ^a^* |
| *Diabetes mellitus, % (n)* | *16.3 (25)* | *22.2 (6)* | *15.1 (19)* | *.381 ^a^* |
| *Obesity, % (n)* | *28.8 (44)* | *48.1 (13)* | *24.6 (31)* | *.016 ^a^* |
| *COPD, % (n)* | *2.0 (3)* | *3.7 (1)* | *1.6 (2)* | *.430 ^a^* |
| *Asthma, % (n)* | *7.8 (12)* | *7.4 (2)* | *7.9 (10)* | *1.000 ^a^* |
| *Chronic kidney injury, % (n)* | *13.1 (20)* | *18.5 (5)* | *11.9 (15)* | *.339 ^a^* |
| *Baseline medication* | *56.2 (86)* | *81.5 (22)* | *50.8 (64)* | *.002 ^a^* |
| *Immunosuppressants, % (n)* | *9.2 (14)* | *11.1 (3)* | *8.7 (11)* | *.708 ^a^* |
| *Beta-blockers, % (n)* | *30.7 (47)* | *63.0 (17)* | *23.8 (30)* | *<.001 ^a^* |
| *ACE-/AT-1-inhibitors, % (n)* | *30.1 (46)* | *44.4 (12)* | *27.0 (34)* | *.061 ^a^* |
| *Insulin, % (n)* | *7.2 (11)* | *14.8 (4)* | *5.6 (7)* | *.093 ^a^* |
| *Metformin, % (n)* | *8.5 (13)* | *3.7 (1)* | *9.5 (12)* | *.698 ^a^* |
| *Statins, % (n)* | *17.0 (26)* | *29.6 (8)* | *14.3 (18)* | *.049 ^a^* |
| *Diuretics, % (n)* | *17.6 (27)* | *33.3 (9)* | *14.3 (18)* | *.022 ^a^* |
| *ASS, % (n)* | *15.7 (24)* | *40.7 (11)* | *10.3 (13)* | *<.001 ^a^* |
| ***CT scan, % (n)*** | *83.0 (127)* | *92.6 (25)* | *81.0 (102)* | *.081 ^a^* |
| *CT scan with CM, % (n)* | *33.3 (51)* | *37.0 (10)* | *32.5 (41)* | *.649 ^a^* |
| *CM volume in CT scan, % (ml) ^c^* | *70 (70-70)* | *70 (70-70)* | *70 (70-70)* | *.648 ^b^* |
| ***Further information regarding the composite endpoint*** | | |  |  |
| *Days until admission to ICU ^c^* | *0 (0-2)* |  |  |  |
| *Days until death ^c^* | *14 (8-24.5)* |  |  |  |

^a^ Fisher’s exact test

^b^ Mann-Whitney-U

^c^ Median (interquartile range)
