# Supplementary material for: Bioactive adrenomedullin and interleukin-6 in COVID-19: potential biomarkers of acute kidney injury and critical illness
Source: BMC Nephrol. 2024 Feb 9;25:52. doi: 10.1186/s12882-024-03486-1 (PMC10858491; doi:10.1186/s12882-024-03486-1)
Supplement: Supplementary file 2 — Additional file 2: Supplementarly Table S2. Further Baseline Characteristics regarding hospitalized patients. [file 12882_2024_3486_MOESM2_ESM.docx]

**Supplementarly Table S2: Further Baseline Characteristics regarding hospitalized patients**

|  | *Hospitalized patients* | *AKI* | *No AKI* | *p AKI vs no AKI* |
| --- | --- | --- | --- | --- |
| *n* | *128* | *22* | *106* |  |
| *Symptoms* |  |  |  |  |
| *Cough, % (n)* | *60.2 (77)* | *54.5 (12)* | *61.3 (65)* | *.635 ^a^* |
| *Dyspnea, % (n)* | *60.9 (78)* | *77.3 (17)* | *57.5 (61)* | *.098 ^a^* |
| *Fever, % (n)* | *58.6 (75)* | *50.0 (11)* | *60.4 (64)* | *.476 ^a^* |
| *Chills, % (n)* | *32.0 (41)* | *22.7 (5)* | *34.0 (36)* | *.452 ^a^* |
| *Sniffles, % (n)* | *11.7 (15)* | *0 (0)* | *14.2 (15)* | *.072 ^a^* |
| *Fatigue, % (n)* | *78.9 (101)* | *90.9 (20)* | *76.4 (81)* | *.160 ^a^* |
| *Anosmia, % (n)* | *15.6 (20)* | *13.6 (3)* | *16.0 (17)* | *1.000 ^a^* |
| *Dysgeusia, % (n)* | *34.4 (44)* | *27.3 (6)* | *35.8 (38)* | *.622 ^a^* |
| *Pre-existing diseases* |  |  |  |  |
| *Coronary artery disease, % (n)* | *10.2 (13)* | *22.7 (5)* | *7.5 (8)* | *.048 ^a^* |
| *Chronic heart failure, % (n)* | *3.1 (4)* | *9.1 (2)* | *1.9 (2)* | *.136 ^a^* |
| *Arterial hypertension, % (n)* | *43.8 (56)* | *68.2 (15)* | *38.7 (41)* | *.017 ^a^* |
| *Diabetes mellitus, % (n)* | *18.8 (24)* | *22.7 (5)* | *17.9 (19)* | *.561 ^a^* |
| *Obesity, % (n)* | *30.5 (39)* | *40.9 (9)* | *28.3 (30)* | *.309 ^a^* |
| *COPD, % (n)* | *2.3 (3)* | *0 (0)* | *2.8 (3)* | *1.000 ^a^* |
| *Asthma, % (n)* | *9.4 (12)* | *9.1 (2)* | *9.4 (10)* | *1.000 ^a^* |
| *Chronic kidney injury, % (n)* | *14.8 (19)* | *31.8 (7)* | *11.3 (12)* | *.022 ^a^* |
| *Baseline medication* | *60.9 (78)* | *86.4 (19)* | *55.7 (59)* | *.008 ^a^* |
| *Immunosuppressants, % (n)* | *10.2 (13)* | *18.2 (4)* | *8.5 (9)* | *.236 ^a^* |
| *Beta-blockers, % (n)* | *32.0 (41)* | *63.6 (14)* | *25.5 (27)* | *<0.001 ^a^* |
| *ACE-/AT-1-inhibitors, % (n)* | *30.5 (39)* | *50.0 (11)* | *26.4 (28)* | *.041 ^a^* |
| *Insulin, % (n)* | *8.6 (11)* | *22.7 (5)* | *5.7 (6)* | *.022 ^a^* |
| *Metformin, % (n)* | *9.4 (12)* | *4.5 (1)* | *10.4 (11)* | *.690 ^a^* |
| *Statins, % (n)* | *17.2 (22)* | *27.3 (6)* | *15.1 (16)* | *.212 ^a^* |
| *Diuretics, % (n)* | *20.3 (26)* | *45.5 (10)* | *15.1 (16)* | *.003 ^a^* |
| *ASS, % (n)* | *17.2 (22)* | *31.8 (7)* | *14.2 (15)* | *.062 ^a^* |
| ***CT scan, % (n)*** | *90.6 (116)* | *90.9 (20)* | *90.6 (96)* | *1.000 ^a^* |
| *CT scan with CM, % (n)* | *37.5 (48)* | *36.4 (8)* | *37.7 (40)* | *1.000 ^a^* |
| *CM volume in CT scan, % (ml) ^c^* | *70 (70-70)* | *70 (70-70)* | *70 (70-70)* | *.734 ^b^* |
| ***Further information regarding AKI*** | | |  |  |
| *Days until development of AKI ^e^* | *5 (2.75-10)* |  |  |  |

^a^ Fisher’s exact test

^b^ Mann-Whitney-U

^c^ Median (interquartile range)
